# Association of Serum FAM19A5 with Cognitive Impairment in Vascular Dementia

**DOI:** 10.1155/2020/8895900

**Published:** 2020-08-01

**Authors:** Juan Li, Shoulin Li, Yihong Song, Wei Zhou, Xiaohao Zhu, Suo Xu, Yihong Ma, Chunlin Zhu

**Affiliations:** ^1^Department of Neurology, The First People's Hospital of Lianyungang/ Department of Neurology, The First Affiliated Hospital of Kangda College of Nanjing Medical University, Lianyungang, Jiangsu Province 222000, China; ^2^Department of Emergency Medicine, The First People's Hospital of Lianyungang/ Department of Neurology, The First Affiliated Hospital of Kangda College of Nanjing Medical University, Lianyungang, Jiangsu Province 222000, China; ^3^Department of Neurology, Graduate School of Medical Sciences, Kumamoto University, Kumamoto 860-8556, Japan

## Abstract

**Objective:**

Family with sequence similarity 19 member A5 (FAM19A5), a novel chemokine-like peptide, is a secreted protein mainly expressed in the brain. FAM19A5 was recently found to be involved in a variety of neurological diseases; however, its correlation with vascular dementia (VaD) remains unclear. The aim of the study is to explore the association between serum FAM19A5 and cognitive impairment in subjects with VaD.

**Method:**

136 VaD subjects and 81 normal controls were recruited in the study. Their demographic and clinical baseline data were collected on admission. All subjects received Mini-Mental State Examination (MMSE) evaluation, which was used to test their cognitive functions. A sandwich enzyme-linked immunosorbent assay (ELISA) was applied to detect the serum levels of FAM19A5.

**Results:**

No significant differences were found between the two groups regarding the demographic and clinical baseline data (*p* > 0.05). The serum FAM19A5 levels were significantly higher compared to normal controls (*p* < 0.001). The Spearman correlation analysis indicated that serum FAM19A5 levels and MMSE scores have a significant negative correlation in VaD patients (*r* = −0.414, <0.001). Further multiple regression analysis indicated that serum FAM19A5 levels were independent risk predictors for cognitive functions in VaD (*β* = 0.419, *p* = 0.031).

**Conclusion:**

The serum FAM19A5 level of VaD patients is significantly increased, which may serve as a biomarker to predict cognitive function of VaD.

## 1. Introduction

Vascular dementia (VaD) is defined as a syndrome with different degrees of cognitive function and memory loss, which may range from mild deficits to severe dementia attributed to impaired blood flow to the brain [1, 2]. VaD is the second commonest subtype of dementia after Alzheimer's disease (AD) and has gained more and more attention in recent years [3, 4]. There are no less than 50 million people suffering from dementia globally; this number is predicted to have a threefold increase by 2050 [5, 6]. Growing with age, the risk of VaD almost doubled every 3–5 years [7]. With the increase in human life expectancy and the advent of aging, VaD has emerged as one of the leading health problems in society [8]. Though much progress has been made over the past years, the pathogenesis underlying VaD is still not fully understood, and there are also no effective drugs or other licensed treatments for VaD.

The family with sequence similarity 19 (FAM19A5), first discovered in 2004, was also named TAFA5. FAM19A5 and a member of the CC-chemokine family (MIP-1*α*) were predicted to be distant relatives. Thus, FAM19A5 has recently been described as a novel chemokine-like peptide [9]. As a secretory protein, FAM19A5 is mainly expressed in some specific regions of the brain and adipose tissues with very low expression in other peripheral organs [10]. The FAM19A genes encode more than 100 amino acid proteins at fixed positions characterized by conserved cysteine residues, which are postulated to function as brain-specific chemokines to regulating immune and nervous cells [11].

Though the FAM19A5 protein has been considered to be related to a variety of neurological diseases, little data about its functions in VaD is available. Therefore, the purpose of our study was to explore the relationship between serum FAM19A5 levels and the cognitive function of VaD patients. If the relationship between FAM19A5 and VaD is confirmed, it will have important scientific and clinical implications.

## 2. Materials and Methods

### 2.1. Subjects

Eventually, 136 VaD subjects and 81 normal controls were recruited from the Department of Neurology, The First People's Hospital of Lianyungang/Department of Neurology, The First Affiliated Hospital of Kangda College of Nanjing Medical University. The diagnosis of VaD was on the basis of DSM-V and NINDS-AIREN (National Institute for Neurological Disorders and Stroke, NINDS-AIREN) by attending physician of neurology [12, 13]. Exclusion criteria for recruitment in the study were as follows: [1] subjects suffering from acute cerebral infarction or cerebral embolism; [2] subjects with mental disorders or other types of dementia such as AD; [3] subject with autoimmune disease or infectious disease; [4] subjects with brain trauma or alcohol and drug abuse; and [5] subject has a history of cancer or other serious organic diseases. All the subjects' demographic and clinical baseline information including age, gender, education levels, blood pressure, body mass index (BMI), blood glucose, and lipid index were collected and recorded. The research was authorized by the Ethics Committee of The First People's Hospital of Lianyungang/The First Affiliated Hospital of Kangda College of Nanjing Medical University. The procedures of our study were performed according to the Helsinki declaration. The written approval consents were signed before the study.

### 2.2. Measurement of Serum FAM19A5 Levels

All subjects were fasted for more than 8 hours, and blood samples were collected via the cubital vein the morning after admission. All blood samples were centrifuged at 1200g for 15 minutes and subjected to the determination of serum FAM19A5 levels. Serum FAM19A5 levels of all subjects were tested by a sandwich enzyme-linked immunosorbent assay (ELISA) kits (ProteinTech Group, Wuhan, China) as described previously [14, 15]. The other biochemical parameters were tested using an automatic blood cell analyzer (Hitachi, Tokyo, Japan).

### 2.3. Cognitive Function Testing

Mini-Mental State Examination (MMSE) is a widely used cognitive evaluation tool in the world with 100% sensitivity and 71.4 specificity [16, 17]. The highest score on the MMSE scale is 30, mainly evaluating orientation, writing, registration, visual construction, recording, and other items related to cognitive function. Cognitive impairment is defined as a score of less than 24 by the investigators, who were blinded to the subjects' baseline clinical characteristics [18].

### 2.4. Data Analysis

Quantitative variables were expressed as mean ± SD or percentage. The independent two-sample *t* test was used to compare the differences between continuous variables conforming to normal distribution. The chi-square test was used for categorical variables. The Spearman correlation analysis was used to analyze the relationship between demographic and clinical characteristics and the cognitive function of VaD patients. Multivariate linear regression analysis determined the predictive value of demographic and clinical characteristics for the cognitive function of VaD patients. A two-tailed *p* < 0.05 was considered statistically significant. The SPSS 23.0 statistical software (SPSS Inc., IL, USA) was used to analyze the data.

## 3. Results

### 3.1. Demographic and Clinical Baseline Characteristics

**Figure 1:**
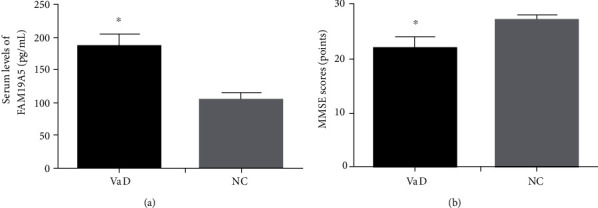
Serum levels of FAM19A5 and MMSE scores in VaD and NC. (a) Serum levels of FAM19A5. (b) MMSE scores. Compared with NC, ∗*p* < 0.001. VaD: vascular dementia; NC: normal controls.

A total of 196 patients presented to the Neurology department at the Lianyungang First People's Hospital for VaD between March 2017 and March 2020. 136 of them and 81 normal controls were found to fulfill the criteria for further analysis. The demographic and clinical baseline data of VaD subjects and normal controls are shown in Table 1. The two groups did not have significant difference in age, gender, educational, SBP, DBP, BMI, FBG, HbA1c, TC, HDL-C, LDL-C, or TG (*p* > 0.05). As shown in Figure 1, there was a significant difference between serum FAM19A5 levels and MMSE scores (*p* < 0.001).

### 3.2. Spearman's Correlation Analysis

Spearman's correlation analysis identified the correlation between demographic and clinical characteristics and MMSE scores, and the results are shown in Table 2. Spearman's correlation analysis showed that the levels of serum FAM19A5 were negatively correlated with MMSE scores in VaD patients, and the correlation was significantly (*r* = −0.424, *p* < 0.05). However, there was no significant correlation in our current study among serum FAM19A5 levels and demographic and statistical characteristics of VaD patients such as age, gender, educational, SBP, DBP, BMI, FBG, HbA1c, TC, HDL-C, LDL-C, and TG (*p* > 0.05).

### 3.3. Multiple Regression Analysis

The results of multiple linear regression analysis among demographic and clinical baseline characteristics, MMSE scores, and serum FAM19A5 levels of VaD subjects are shown in Table 3. The results of multiple linear regression analysis demonstrated that serum FAM19A5 level is an independent predictive risk marker for cognitive function in VaD patients. After adjusting for demographic and clinical baseline characteristics such as age, gender, educational, SBP, DBP, BMI, FBG, HbA1c, TC, HDL-C, LDL-C, and TG, the independent value of serum FAM19A5 level for VaD patients' cognitive function is still significant (*β* = 0.419, *p* = 0.031).

## 4. Discussion

This study is investigating the association between cognitive functions and FAM19A5 levels in patient and control groups. We found that patients with VaD had higher serum FAM19A5 levels and lower MMSE scores than normal controls. We also found that in VaD patients, serum levels of FAM19A5 were significantly negatively correlated with MMSE scores. Serum FAM19A5 levels were found to be related to cognitive functions in VaD, whereas other demographic and statistical characteristics were not related to cognitive functions in VaD. This association was independent of the interference factors of age, gender, educational, SBP, DBP, BMI, FBG, HbA1c, TC, HDL-C, LDL-C, and TG. As far as we know, we demonstrated for the first time that serum FAM19A5 level was an independent risk factor for cognitive impairment in VaD patients.

FAM19A5 is one of the five highly homologous family members of family with sequence similarity 19 (FAM19A) [19]. The secreted proteins discovered by a new database search strategy are mainly expressed in the adipose tissue and central nervous system [20]. According to reports, FAM19A5 is mainly expressed in the hippocampus, hypothalamic paranucleus, and suprabranchial nucleus in rats, while in mice, the main expression site is in the hippocampus [21, 22]. Kong and his team first proved that FAM19A5 is a secreted protein, and the 43 amino acids starting at the N-terminus are its main signal peptides [11]. Although FAM19A5 has been discovered for more than ten years, its function has not been fully elucidated.

Recently, FAM19A5 has been found to be associated with a variety of diseases and disorders. Han and his colleagues explore the significance of a novel chemokine-like peptide (FAM19A5), which was associated with the presence of depressive symptoms and might be a biomarker of neuroinflammation and neurodegeneration for major depressive disorder [23]. In addition, researchers from Korea University confirmed that serum FAM19A5 may be a novel biomarker for cardiovascular metabolic diseases, which was significantly correlated to various human metabolic and vascular risk factors [9]. Proteomics research demonstrates that FAM19A5 has a potential to serve as a novel biomarker for differentiating cholangiocarcinoma from benign biliary tract diseases [24]. In parallel, a Chinese study showed that FAM19A5 had significant effects on gastric cancer progression and may serve as a potential therapeutic target for gastric cancer [25]. However, the correlation between FAM19A5 and VaD has not been reported so far.

Emerging evidence has indicated that FAM19A family is involved in the pathological mechanism of cognition. FAM19A2 knockout mice exhibited decline in short-term and long-term memory via novel object recognition test and decline in spatial learning and memory via the Morris water maze (MWM) test [26, 27]. However, FAM19A1 knockout mice only exhibited reduced anxiety and sensitivity to pain, and spatial learning and exploration are preserved [28, 29]. Interestingly, recent studies have shown that FAM19A5 is involved in the development of the nervous system. A Korean study shows FAM19A5 plays a vital role at an early stage of nervous system development, and its functions are critical to the generation of stem cells in the adult brain such as neural stem cells and oligodendrocyte precursor cells [22]. Not only in animal experiments, the correlation between FAM19A5 and neurodevelopment in clinical studies has also been found [30, 31]. More recent research shows that selective overexpression of FAM19A5 in the mouse hippocampus can alleviate chronic stress-related spatial learning and memory impairment [32]. All the above studies indicate that the FAM19A family including FAM19A5 plays an important role in cognition. However, the level of FAM19A5 in different neurological diseases is fluctuating. The mechanism by which FAM19A5 participates in cognitive impairment is complex and needs further elucidation.

The current study has some limitations. First of all, this study has a relatively small sample size. Secondly, the subjects are all Chinese, and the results may not apply to other ethnic groups. Thirdly, we did not investigate the FAM19A5 levels in other types of dementias such as Alzheimer's dementia and hydrocephalus with normal intracranial pressure. Thus, the correlation between serum FAM19A5 levels and cognitive impairment may not be unique to VaD. At last, we just did a cross-sectional study of serum FAM19A5 levels and did not follow up longitudinally. However, the advantage of our study is that this is the first report of the association between FAM19A5 levels and cognitive impairment in VaD.

## 5. Conclusions

In short, the main findings of our research are that VaD subjects have a significantly lower serum level of FAM19A5 compared to normal controls. Our study revealed that serum FAM19A5 plays an important role for the first time in the regulation of cognitive impairment in VaD. We look forward to a larger multicenter study in the future to further confirm the correlation between serum FAM19A5 levels and cognitive functions in VaD subjects. Clarifying the underlying pathological mechanisms of FAM19A5 involved in the onset of VaD will have important clinical significance.

## Figures and Tables

**Table 1:** Baseline characteristics and serum FAM19A5 levels.

	VaD (*n* = 136)	NC (*n* = 81)	*p*
Age, years	69.8 ± 7.1	70.5 ± 6.9	0.479
Male, *n* (%)	77 (56.7)	47 (58.0)	0.761
Education, years	7.2 ± 1.9	7.4 ± 2.2	0.481
BMI, kg/m [2]	24.8 ± 1.7	24.6 ± 1.5	0.383
SBP, mmHg	145.2 ± 10.4	143.9 ± 9.6	0.361
DBP, mmHg	93.3 ± 7.2	92.8 ± 7.5	0.627
FBG, mmol/L	6.38 ± 0.72	6.41 ± 0.81	0.777
HbA1c, mmol/L	6.36 ± 0.70	6.38 ± 0.77	0.845
TC, mmol/L	4.47 ± 0.65	4.52 ± 0.62	0.578
HDL-C, mmol/L	1.28 ± 0.16	1.25 ± 0.13	0.154
LDL-C, mmol/L	2.73 ± 0.21	2.69 ± 0.26	0.216
TG, mmol/L	1.64 ± 0.20	1.61 ± 0.24	0.323
FAM19A5, pg/mL	186.3 ± 17.9	103.2 ± 11.6	<0.001
MMSE	21.8 ± 2.1	27.0 ± 0.9	<0.001

VaD: vascular dementia; NC: normal controls; SBP: systolic blood pressure; DBP: diastolic blood pressure; BMI: body mass index; FBG: fasting blood glucose; HbA1c: hemoglobin A1c; TC: total cholesterol; HDL-C: high-density lipoprotein cholesterol; LDL-C: low-density lipoprotein cholesterol; TG: triglycerides; FAM19A5: family with sequence similarity 19 member A5; MMSE: Mini-Mental State Examination.

**Table 2:** Spearman's correlation analysis of MMSE scores and various parameters in VaD patients.

	MMSE	Scores
	*r*	*p*
FAM19A5, pg/mL	-0.424	<0.001

VaD: vascular dementia; FAM19A5: family with sequence similarity 19 member A5; MMSE: Mini-Mental State Examination.

**Table 3:** Regression analyses between MMSE and characteristics of VaD patients.

	Standardized	Coefficients	95% CI for B	
	*β*	*p*	Lower bound	Upper bound
FAM19A5	0.419	0.031	1.239	1.708

MMSE: Mini-Mental State Examination; VaD: vascular dementia; FAM19A5: family with sequence similarity 19 member A5.

## Data Availability

The data used to support the findings of this study are available from the corresponding author upon request.
